# Advanced maternal age and childhood allergy: marker or mechanism?

**DOI:** 10.3389/falgy.2026.1825315

**Published:** 2026-05-04

**Authors:** Cristiana Indolfi, Angela Klain, Carolina Grella, Giulio Dinardo, Salvatore Cascone, Michele Miraglia del Giudice

**Affiliations:** Department of Woman, Child and General and Specialized Surgery, University of Campania Luigi Vanvitelli, Naples, Italy

**Keywords:** advanced maternal age, allergy prevention, childhood allergy, children, early-life immune development

## 1 Introduction

Over the past three decades, the epidemiology of allergic diseases has been interpreted through shifting conceptual frameworks, moving from the classic hygiene hypothesis to more complex ecological and developmental models of immune programming (1). Within this evolving landscape, parental age, a demographic factor frequently examined in studies of early-life determinants of health (2–4), has intermittently emerged as a potential determinant of childhood allergy risk, yet findings have been inconsistent across populations and time periods. The recent large prospective cohort study by Yamamoto-Hanada et al. (5) reporting an inverse association between advanced maternal age and early childhood allergic outcomes reopens this discussion and invites a more nuanced interpretation of what parental age truly represents in contemporary societies.

## 2 Interpreting the association

At first glance, the finding that older maternal age is associated with lower risk of certain allergic outcomes may appear counterintuitive, particularly in light of earlier studies suggesting either no association or even increased risk with advanced parental age (6). Notably, Zhang et al. (6), in a large cross-sectional study of 15,976 children in Shanghai, reported that parental age ≥25 years was associated with an increased risk of most childhood allergic diseases, with a stronger effect observed for paternal age. These findings contrast with those of Yamamoto-Hanada et al., highlighting important heterogeneity across populations. Several factors may account for these discrepancies. First, differences in study design may play a role, as Zhang et al. conducted a cross-sectional analysis, whereas Yamamoto-Hanada et al. used a prospective birth cohort, which may better capture temporal relationships and reduce recall bias. Second, the definition of “advanced” parental age differs substantially (≥25 years vs. ≥35 years), potentially reflecting distinct biological and social contexts. Third, environmental and cultural differences between Chinese and Japanese populations, including lifestyle, pollution exposure, healthcare practices, and infant feeding behaviors, may influence both parental age patterns and allergy risk. Finally, variation in adjustment for confounding factors, such as socioeconomic status, parity, and breastfeeding practices, may further contribute to the observed divergence.

The developmental origins of health and disease (DOHaD) paradigm have emphasized the critical importance of prenatal and early postnatal environments in shaping immune trajectories (7). During this window, immune maturation is highly sensitive to nutritional exposures, microbial encounters, barrier integrity, and inflammatory signals. Maternal age may influence these exposures indirectly through differences in socioeconomic status, health literacy, healthcare engagement, and parenting practices. In many high-income settings, delayed parenthood is associated with higher educational attainment, more stable income, planned pregnancies, and earlier access to prenatal care. These factors may, in turn, shape infant feeding patterns, eczema management, vaccination uptake, and adherence to preventive guidelines (8, 9).

The Japanese context in which the cohort by Yamamoto-Hanada et al. (2) was conducted is particularly informative. Japan has one of the highest mean maternal ages at childbirth globally, currently exceeding 32 years (10). This reflects a broader demographic trend toward delayed parenthood. At the same time, reported prevalence rates of several childhood allergic conditions remain comparatively lower than in parts of Asia and in Central and South America, where mean maternal age remains below 27 years (6, 11–13). It is notable that in several Central and South American countries, younger maternal age distributions coexist with relatively high prevalence of allergic rhinitis and other atopic conditions, as documented in ISAAC phase three and subsequent regional surveys (13–15). These geographic contrasts are directionally consistent with the inverse association reported in the Japanese cohort, suggesting that maternal age may index broader sociodemographic transitions rather than exerting a direct biological effect. Ecological comparisons must be interpreted cautiously due to heterogeneity in methodology, case definitions, diagnostic awareness, and environmental exposures. Therefore, such observations cannot be used to infer individual-level relationships between maternal age and allergic outcomes, and should be viewed as hypothesis-generating rather than confirmatory.

Historically, advanced parental age was often discussed within the framework of the hygiene hypothesis, first articulated by Strachan in 1989 (16). The hypothesis proposed that reduced exposure to infectious agents and siblings in early life increased susceptibility to allergic disease. Older parents were presumed more likely to adopt protective, sanitation-oriented child-rearing practices, limit microbial exposures, delay daycare entry, and maintain highly controlled domestic environments. Under that paradigm, one might have anticipated increased allergic risk among children of older parents.

However, the hygiene hypothesis has evolved substantially. What initially emerged as a unidimensional explanation centered on reduced microbial exposure has progressively transitioned into a multidimensional framework that integrates environmental, immunologic, epigenetic, and microbial determinants of immune development (17). Contemporary models increasingly rely on multi-omic approaches, including genomics, epigenomics, transcriptomics, metabolomics, and microbiomics, to characterize how early-life exposures shape immune trajectories in a systems-level manner (17). Rather than attributing allergic disease to simple “excess hygiene,” current evidence suggests that immune maturation reflects complex interactions between host genetics, microbial ecology, epithelial barrier integrity, metabolic signaling, and environmental biodiversity (18, 19). Simultaneously, the preventive approach to food allergy has undergone a profound transformation. Landmark randomized trials such as LEAP and EAT demonstrated that early introduction of peanut and other allergenic foods can significantly reduce the development of food allergy in high-risk infants (20, 21). These findings prompted updated guidelines recommending early and sustained allergen exposure rather than delayed introduction (22). In parallel, increasing attention has been devoted to the role of skin-barrier integrity in preventing epicutaneous sensitization (23). Early emollient therapy and proactive eczema management have been proposed as strategies to reduce atopic dermatitis and possibly food sensitization, although results across trials have been mixed (24, 25). What is clear, however, is that contemporary allergy prevention no longer centers on avoidance but on strategic exposure and barrier support.

## 3 Discussion

Within this modern preventive framework, maternal age may correlate with differential uptake of updated recommendations. Older parents, on average, may have greater interaction with healthcare systems, higher exposure to public health messaging, and more opportunities to implement early allergen introduction, appropriate eczema management, and evidence-based feeding practices. The translation of trial evidence into real-world practice has not been uniform across populations (26, 27). Health literacy, cultural norms, access to pediatric care, and trust in medical institutions all shape adoption of preventive strategies. Thus, maternal age may reflect disparities in implementation rather than intrinsic immunobiological differences.

Breastfeeding provides an additional example of the complexity of mediating pathways. The association between breastfeeding and allergic outcomes remains heterogeneous and context-dependent (28). Zhang et al. (6) suggested that breastfeeding may modify the association between parental age and childhood allergic disease, underscoring the interconnected nature of early-life exposures. Advanced maternal age is frequently associated with higher breastfeeding initiation rates and longer duration in many high-income settings, potentially influencing immune development through microbiota modulation and immune-active components in breast milk.

Structural determinants must also be considered. Delayed parenthood is often linked to urban residence, smaller family size, and more stable housing conditions (29). These characteristics may influence indoor allergen exposure, air pollution burden, pet ownership, and access to green space. The relationship between urbanization and allergic disease is itself complex, shaped by environmental biodiversity, lifestyle factors, and socioeconomic gradients (30). Parental age is embedded within these broader societal patterns. From a biological standpoint, the mechanisms linking advanced parental age to offspring allergic risk remain incompletely understood, but several plausible pathways merit consideration. First, age-related epigenetic modifications in both oocytes and sperm, particularly at imprinted loci, have been associated with altered placental development and function, which could in turn influence early immune programming (31). Second, maternal age has been linked to differences in vaginal and gut microbiota composition, potentially affecting neonatal microbial colonization and the maturation of the infant immune system (32, 33). Third, increasing maternal age may reflect cumulative immunological experience, including a broader T-cell repertoire and more diverse transplacental IgG transfer, which could shape early-life immune responses and tolerance development (34).

These hypotheses are supported by a growing body of literature indicating that epigenetic mechanisms play a central role in mediating early-life environmental influences on allergic disease risk. DNA methylation changes at immune-regulatory loci, including *IFNG*, *IL4*, *FOXP3*, and *GATA3*, can influence Th1/Th2 balance and regulatory T-cell differentiation, key processes underlying allergic sensitization (35). In addition, microRNAs such as miR-155 and miR-21 have been implicated in modulating inflammatory pathways and IgE responses, further linking epigenetic regulation to allergy development. These epigenetic signatures are particularly sensitive to environmental exposures during prenatal and early postnatal life. Consistently, Acevedo et al. highlight that perinatal exposures, including maternal diet (e.g., folate, vitamin D, and polyunsaturated fatty acids), as well as microbiome-derived metabolites such as short-chain fatty acids (butyrate, acetate, propionate), can induce epigenetic modifications in immune cells during critical developmental windows (36). These signals influence histone acetylation and DNA methylation patterns that regulate neonatal T-cell polarization, promote regulatory T-cell (Treg) function, and enhance immune tolerance, thereby reducing the risk of allergic sensitization. Moreover, microbial colonization at birth, shaped in part by maternal microbiota, further reinforces these epigenetic and immunological pathways (36).

In parallel, age-related epigenetic alterations in gametes provide an additional layer of biological plausibility. Advanced parental age was demonstrated to be associated with altered DNA methylation at imprinted genes such as *H19*, *IGF2*, and *KCNQ1OT1*, which are critical for placental growth and nutrient transfer (31). Dysregulation at these loci may affect placental endocrine and barrier functions, thereby modifying the intrauterine environment and influencing fetal immune programming. Such changes could plausibly impact early immune trajectories, although direct links with reduced allergic risk remain to be established.

At present, however, direct evidence connecting these biological processes to a reduced risk of allergic outcomes is limited and not always consistent. Rather than supporting a single mechanistic explanation, the available data suggest that parental age may act as a proxy for a constellation of biological and environmental influences operating during critical windows of immune development. The prospective design and standardized outcome assessment in the Japanese cohort strengthen internal validity (5). Nevertheless, residual confounding by socioeconomic status, parental atopy, daycare attendance, sibling number, and environmental exposures cannot be fully excluded. Parental atopy in particular is a strong predictor of offspring allergic risk and may correlate with reproductive timing. Without detailed mediation analyses, distinguishing direct biological effects from correlated behavioral and environmental pathways remains challenging.

While this discussion has focused on maternal age, paternal age may also independently influence offspring allergy risk through sperm epigenetic changes and *de novo* mutations. In addition, paternal age may capture distinct socioeconomic and environmental factors (31, 35). Notably, in the Shanghai cohort, paternal age showed a stronger association with allergic outcomes than maternal age (6).

In addition to confounding, the possibility of reverse causation or selection bias should also be considered. Women with allergic disease may experience delayed childbearing due to disease burden, treatment, or related lifestyle factors, potentially linking maternal atopy to both age at delivery and offspring allergy risk. This could partially contribute to the observed associations and complicate causal interpretation.

**Figure 1:**
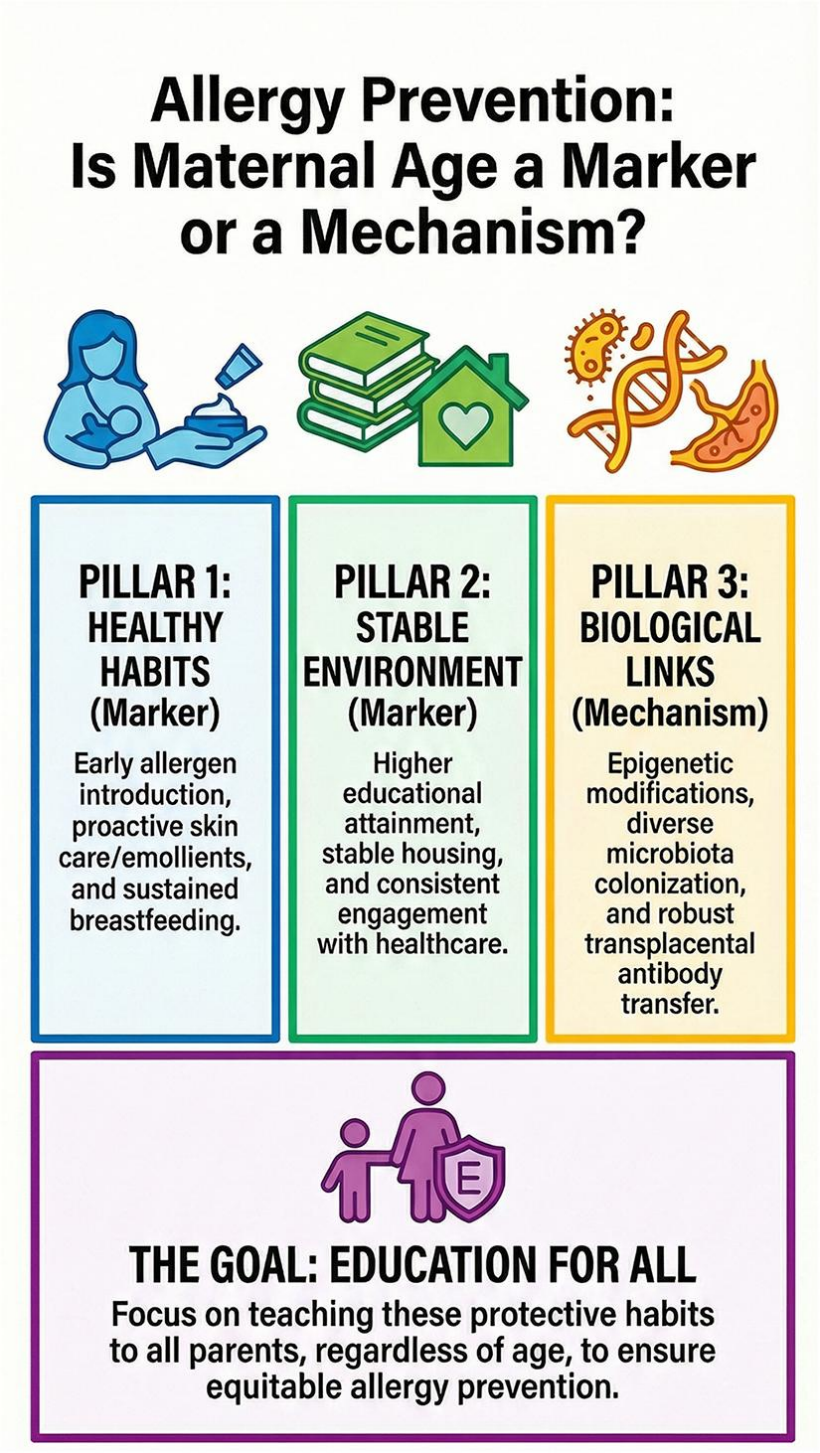
Conceptual framework of parental age as a marker or mechanism in childhood allergy risk.

From a public health perspective, parental age itself is not a modifiable intervention target for allergy prevention. Parental age at the time of a child's birth is primarily a demographic characteristic reflecting reproductive timing and broader social circumstances rather than a factor that can be directly modified through preventive health interventions. Framing advanced maternal age as protective risks misinterpretation and could inadvertently reinforce inequities affecting younger parents. Instead, the actionable message lies in identifying and disseminating the protective behaviors and healthcare interactions that maternal age may proxy. Ensuring equitable access to early allergen introduction guidance, culturally appropriate feeding counseling, proactive eczema care, breastfeeding support, and pediatric follow-up is essential. The first 1,000 days of life remain a critical window for immune education and long-term allergic trajectories (37). During this period, coordinated policies supporting parental leave, healthcare accessibility, health education, and socioeconomic stability may exert far greater influence on allergic outcomes than parental age *per se*. If older parents are currently more likely to implement contemporary prevention strategies, closing that gap across age groups should be a priority. The conceptual framework summarizing the proposed pathways linking parental age to childhood allergic outcomes is illustrated in Figure 1.

Parental age is represented as a central factor influencing childhood allergic outcomes through three interconnected pathways: (1) behavioural factors, including early allergen introduction, breastfeeding, and preventive care; (2) socioeconomic and environmental conditions, such as education, housing stability, and exposure patterns; and (3) biological mechanisms, including epigenetic modifications, microbiota development, and transplacental immune transfer. These pathways converge on early-life immune development, shaping susceptibility to allergic diseases.

In summary, the inverse association between advanced maternal age and early childhood allergic outcomes reported by Yamamoto-Hanada et al. (5) contributes valuable evidence to an evolving field. Rather than viewing maternal age as a direct biological determinant, it may be more appropriate to interpret it as an integrative marker of socioeconomic position, healthcare engagement, and adherence to modern preventive practices. Geographic contrasts, historical shifts in allergy paradigms, and mediation through early-life behaviors all support this interpretation. Future research should prioritize mechanistic mediation analyses and cross-cultural comparisons to disentangle biological from contextual pathways. Ultimately, advancing allergy prevention will depend not on demographic characteristics but on equitable implementation of evidence-based strategies that support optimal immune development for all children.
